# Ranking patients’ non-clinical preferences in referring to specialist physicians in the private sector: a cross-sectional study

**DOI:** 10.1186/s12913-025-13643-3

**Published:** 2025-11-25

**Authors:** Masood Setoodefar, Hamed Tabesh, Kobra Etminani, Mohammad Reza Mazaheri Habibi, Zahra Dabir, Seyed Ali Fatemi Aghda

**Affiliations:** 1 Department of Computer Sciences, Faculty of Engineering, Khayyam University, Mashhad, Iran; 2 Department of Medical Informatics, School of Medicine, Mashhad University of Medical Sciences, Mashhad, Iran; 3 Center for Applied Intelligent Systems Research, Halmstad University, Halmstad, Sweden; 4 Department of Health Information Technology, Varastegan Institute for Medical Sciences, Mashhad, Iran; 5 Fakher Mechatronic Research Center, Kerman University of Medical Sciences, Kerman, Iran; 6 Student Research and Technology Committee, School of Health Management and Information Sciences, Iran University of Medical Sciences, Tehran, Iran; 7 Research Center for Health Technology Assessment and Medical Informatics, School of Public Health, Shahid Sadoughi University of Medical Sciences, Yazd, Iran

**Keywords:** Patient preference, Specialist physicians, Private healthcare, Obstetrics, Gynecology, Patient-centered care

## Abstract

**Background:**

Understanding non-clinical factors that influence how women choose obstetricians and gynecologists (OB/GYNs) is essential for delivering patient-centered care. This study aimed to identify and rank the non-clinical preferences when selecting OB/GYN specialists in the private healthcare sector in Mashhad, Iran.

**Methods:**

This cross-sectional study, conducted from January to February 2018, 462 patients completed a validated 45-item questionnaire (CVI = 0.80, Cronbach’s alpha = 0.88) assessing their non-clinical preferences. Preferences were rated on a 5-point scale and ranked using Friedman’s test. Associations between demographic factors and preferences were analyzed using the Kruskal-Wallis test and ordinal logistic regression.

**Results:**

The highest-rated criteria included physicians’ attentiveness and respect for patients, respectful staff behavior, short waiting times, and ensuring privacy during examinations. The latest important criteria were physician age, university affiliation, and office proximity to patient’s home. Education level, pregnancy experience, and number of prior OB/GYN visits were significantly associated with certain preferences. Multivariate regression revealed that higher education and more prior OB/GYN visits independently predicted greater importance placed on short waiting time and respectful staff behavior.

**Conclusion:**

Beyond clinical competence, non-clinical factors-particularly those related to interpersonal behavior, communication, and privacy-are central to patient-centered care in OB/GYN settings. Recognizing and integrating these preferences into service delivery can strengthen trust, enhance satisfaction, and support ethical, patient-centered care in the private healthcare sector.

## Background

Delivering patient-centered care requires healthcare professionals to consider not only clinical effectiveness but also the values, needs, and preferences of the individuals they serve [1]. Patients prefer to have a trusting and communicative relationship with their physician rather than a business-oriented one [2]. In private obstetrics and gynecology (OB/GYN) practices, where patients often self-refer, understanding these preferences becomes essential to fostering trust, ensuring respectful care, and promoting shared decision-making [3].

Studies have shown that patient decision-making about the quality of services provided by a physician is a multidimensional and complex process [4]. While patients certainly value clinical expertise, research suggests that non-clinical factors also significantly shape their healthcare-seeking behaviors and satisfaction with care. These may include the communication style and demeanor of physicians and staff, the physical environment of the clinic, appointment accessibility, and the perceived responsiveness of care [5–7].

In OB/GYN care where issues of privacy, gender sensitivity, and emotional support are particularly pronounced, these considerations take on even greater importance. Obstetricians and gynecologists offer a variety of specialized procedures to women, including pregnancy and delivery related procedures, cancer screenings, surgeries, after obtaining specialized fellowships [5, 8, 9].

Studies have found that women actively search and compare available options in this field [10]. Women seeking OB/GYN services may navigate multiple options based on recommendations, prior experiences, and their social and cultural contexts. These decisions are often made under conditions of personal vulnerability and require a high degree of trust in the provider [1]. Therefore, understanding the full spectrum of what matters to patients is essential for ensuring ethical, high-quality care that respects their autonomy and dignity.

Physicians who can effectively identify patient preferences can create a robust patient-physician relationship, which can lead to improved patient adherence to treatment and better treatment outcomes, enhancing both patient and physician satisfaction [11]. Dissatisfaction with treatment can lead to patients’ decision to change providers or discontinue treatment entirely [12, 13].

Previous studies have examined individual non-clinical factors affecting provider choice, such as gender preference or waiting time. However, few have comprehensively ranked these criteria within the OB/GYN field or explored how preferences differ by sociodemographic characteristics. This study addresses that gap by identifying and prioritizing the non-clinical factors women consider most important when choosing OB/GYN providers in the private sector in Mashhad, Iran. The objective of this study was to examine the non-clinical factors influencing women’s selection of obstetrician/gynecologists in the private sector, using a patient-centered framework and robust multivariate analysis.

## Methods

### Study design and setting

This cross-sectional study was conducted between January and February 2018 in Mashhad, Iran, the country’s second-largest metropolis and a regional hub for private obstetric and gynecologic care.

### Participants and sampling

Participants were recruited from 63 private OB/GYN offices selected using convenience sampling. On scheduled visit days, all adult female patients (≥18 years old) who were present for an appointment and able to provide informed consent were invited to participate. Individuals in emergency situations or not directly attending the visit (e.g., companions or family members) were excluded.

### Data collection instrument

Data were collected using a structured questionnaire previously developed and validated by the lead researcher (CVI = 0.80; Cronbach’s alpha = 0.88) [14]. The instrument consisted of two main sections: a sociodemographic section that collected data on participants’ age, education level, marital status, employment status, pregnancy history, and number of prior OB/GYN visits; and a preference section comprising 45 items across eight domains of non-clinical care preferences, including *physician characteristics*, *office accessibility and availability*, *communication and interpersonal behavior*, *privacy and confidentiality*, *cost and insurance*, *office environment and facilities*, *recommendations and reputation*, and *access to special diagnostic or para-clinical services*.

Each item was rated on a 5-point Likert scale ranging from *Not Important at All (1)* to *Very Important (5)*. For ease of interpretation, these responses were subsequently grouped into three categories: *Highly Important/Important*, *Neutral (Does not make a difference)*, and *Unimportant/Highly Unimportant*. This approach was based on previous studies using collapsed Likert categories in ranking analyses [15].

### Data analysis

Descriptive statistics were used to summarize participant demographics and response frequencies. Preference items were ranked using Friedman’s test, appropriate for ordinal repeated measures, with all assumptions met. Associations between patient characteristics (*age*, *education*, *marital status*, *pregnancy experience*, and *number of OB/GYN visits*) and preferences were examined using Kruskal-Wallis tests, with significance set at *p* < 0.05.

To assess independent effects of demographics, ordinal logistic regression was conducted for three top-ranked items: *paying attention to the patient*, *short waiting time*, and *respectful staff behavior*. Predictor variables included all demographic factors listed above, with results reported as odds ratios (ORs) and 95% confidence intervals (CIs). All models met proportional odds and model fit criteria. Missing data ( < 1% per item) were handled through listwise exclusion without imputation. The statistical analysis was done using SPSS software version 2

## Results

### Sample characteristics

A total of 480 patients were invited to participate in the study, of whom 462 agreed (response rate: 96.2%). Outlier responses constituted less than 1% per item. Table 1 presents participants’ demographic characteristics. The average age was 30 ± 7.18 years. Over two-thirds of the participants were housewives, and more than 60% of them had visited at least two OB/GYNs. Nearly half (48.5%) attended for pregnancy-related services.

**Table 1:** Sociodemographic characteristics of the participants

Domain	Variable	Sample study data N (%) Count = 462
Age (years)	≤ 19	12(2.6)
	20–29	214(46.3)
	30–39	184(39.7)
	40–49	42(9.1)
	≥ 50	10(2.1)
	missing	1(0.2)
Highest level of education attained	None	6(1.3)
	Below Diploma	70(15.2)
	Diploma	156(33.8)
	Bachelor’s Degree Education	180(39.0)
	Higher than Bachelor’s Degree	46(10.0)
	missing	4(0.9)
Job status	Employee	89(19.3)
	Housewife	323(69.9)
	Out of Home Part-Time	48(10.4)
	missing	2(0.4)
Marital status	Single	18(3.9)
	Married	443(95.9)
	missing	1(0.2)
Pregnancy experience	No	179(38.7)
	Yes	281(60.8)
	missing	2(0.4)
Number of OB/GYN visit so far	I have not visited any ob-gyn before	17(3.7)
	I have visited only one ob-gyn	158(34.2)
	I have visited two or more ob-gyn	287(62.1)
Cause of visit	Pregnancy Care	224(48.5)
	Check-up Annually	122(26.4)
	Female Diseases	115(24.9)
	Missing	1(0.2)

### Descriptive analysis and item ranking

The most frequently prioritized items included physicians’ attentiveness (Q12: 99.0%), respectful behavior during examination (Q13: 97.2%), respectful staff conduct (Q33: 97.3%), and short waiting times (Q26: 94.8%). The least important items were physician’s age (Q3: 45.3%), university affiliation (Q6: 33.0%), and proximity of the office to home (Q21: 47.4%). Table 2 presents full item distributions and rankings. Friedman’s test was applied to rank the importance of all 45 items. Assumptions for ordinal data and repeated measures across items were met.

**Table 2:**
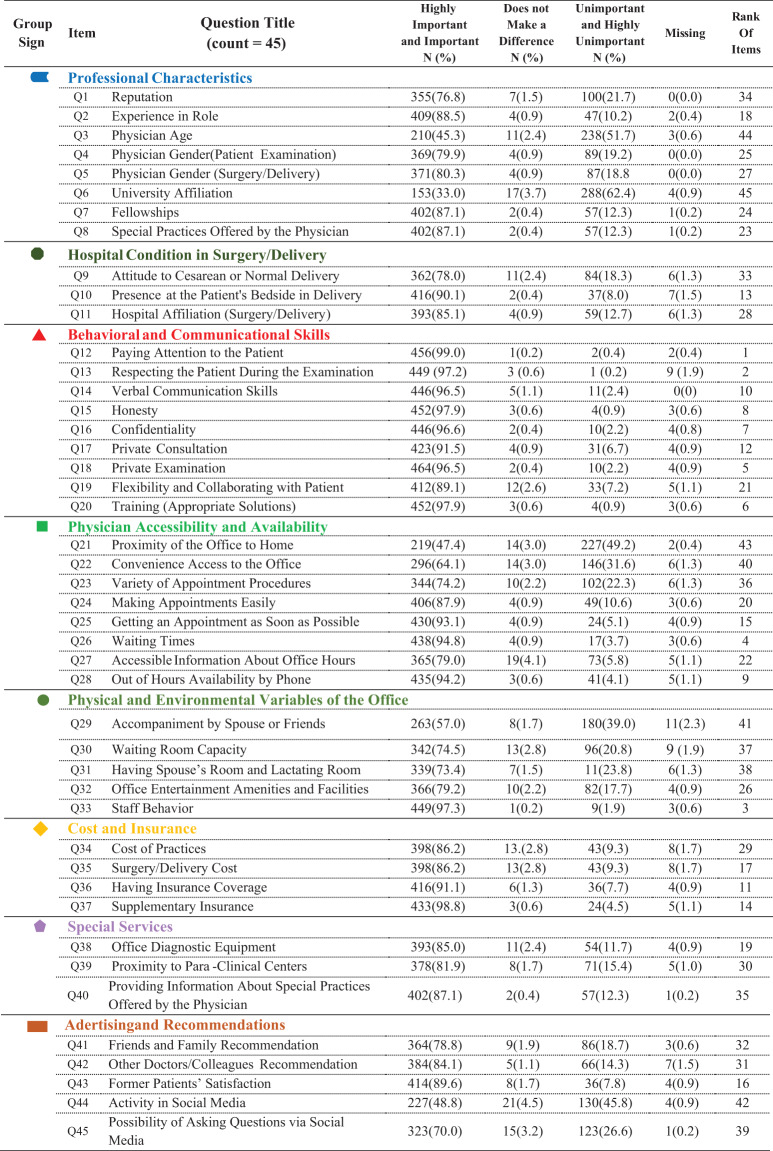
Participants’ response to questionnaire items

### Stratified item rankings

Table 3 presents stratified rankings across patient subgroups (e.g., pregnancy experience, number of OB/GYN visits). Rankings remained consistent across most groups, although slight differences were observed for items such as confidentiality, private consultation, and insurance coverage. The table uses color-coding and graphic symbols to indicate the dimension of each item.

**Table 3:**
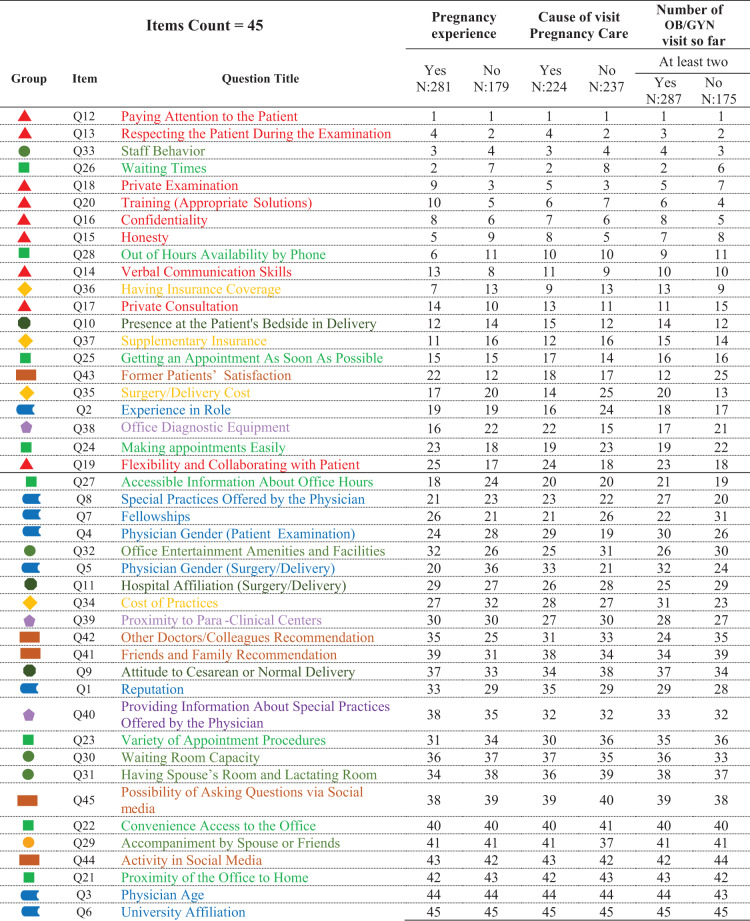
Ranking of questionnaire items

### Bivariate Analysis

Kruskal–Wallis tests revealed significant associations between education level and preferences related to office facilities and insurance (Q31, Q32, Q36, Q37, Q39, Q40). Number of prior OB/GYN visits was also significantly associated with preferences such as paying attention to the patient (Q12), office accessibility (Q27), out-of-hours availability (Q28), and respectful staff behavior (Q33). As expected, these non-parametric tests only identify associations and cannot determine the direction or independent strength of effects. Additional associations are summarized in Table 4.

**Table 4:**
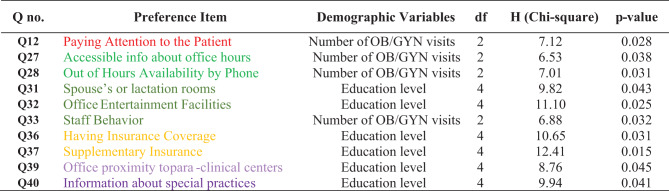
Kruskal–Wallis tests of associations between patient characteristics and preference items

### Multivariate regression analysis

To further evaluate independent predictors, ordinal logistic regression was conducted on three highly rated items. Participants with a bachelor’s degree or higher had significantly greater odds of prioritizing short waiting times (Q26) and respectful staff behavior (Q33). Those with two or more prior OB/GYN visits were more likely to value physician attentiveness (Q12) and waiting time (Q26). Prior pregnancy experience was independently associated with greater importance placed on respectful staff behavior (Q33).These regression findings extend the bivariate results by demonstrating which demographic characteristics independently predict non-clinical care preferences and in which direction (Table 5).

**Table 5:**
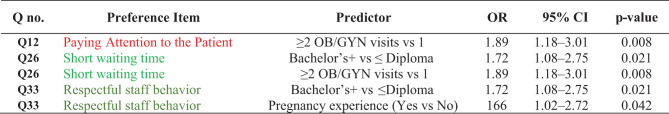
Ordinal logistic regression of demographic predictors of key preferences

## Discussion

This study aimed to identify and rank non-clinical factors that influence women’s decisions when selecting obstetricians and gynecologists in the private healthcare sector. While prior research has emphasized physicians’ clinical skills as central to quality care [16, 17], our findings reinforce that interpersonal, environmental, and accessibility-related dimensions also play critical roles in shaping patients’ evaluations and decisions. These preferences reflect the deeply personal and trust-based nature of OB/GYN care, which often involves sensitive, intimate, and emotionally significant encounters.

Clinical skills are typically assessed based on a physician’s past success rates, as well as their ability to diagnose diseases accurately and choose the most effective treatment methods [18]. However, due to a variety of factors such as economic-cultural conditions and a lack of knowledge and awareness about a physician’s clinical skills, patients often consider other factors when evaluating the quality of services provided and selecting and referring to a physician [3, 5, 9].

The top-rated factors—physician attentiveness, respectful communication, staff behavior, waiting times, and examination privacy—underscore the importance of dignity, respect, and emotional safety in patient experiences. These elements are not ancillary to care quality; rather, they are foundational to building trust and fostering patient engagement. Other highly valued factors—such as clear communication and protection of confidentiality—reflect the principles of patient-centered care, which emphasize transparency, autonomy, and shared decision-making. [11, 19].

Good communication and behavioral skills of physicians, such as verbal skills, manners, appearance, and effective communication, can create trust, improve patient compliance, and result in long-term outcomes and patient satisfaction, according to numerous studies [20, 21].

Shorter waiting times and clear access to appointments were also prioritized, consistent with literature highlighting how system inefficiencies can contribute to frustration and reduce continuity of care [16, 22–24]. As healthcare delivery models increasingly emphasize responsiveness and efficiency, attending to these logistical aspects becomes vital—not only for convenience but for patient reassurance and sustained trust.

The COVID-19 pandemic has underscored the importance of using information technologies (ITs) to schedule patients’ attendance accurately, both in terms of waiting time in the office and after taking the appointment until the visit day. To this end, IT applications such as online appointment systems, SMS reminder systems, patient appointment scheduling systems, and automated phone call reminder systems can improve office efficiency and reduce waiting time. Studies have shown that reminding patients by the secretary can prevent absences and increase regular attendance, leading to greater patient satisfaction [24, 25].

Preserving patient privacy are among the top concerns of patients, particularly in obstetrics and gynecology offices where physicians are in contact with the most private aspects of patients [6, 26, 27]. However, the study revealed that overcrowding may lead to examinations being conducted in groups, which could compromise patient privacy. It is therefore essential to implement appropriate measures to maintain privacy and confidentiality in medical settings.

Notably, availability outside of standard office hours and presence during childbirth were highly valued, suggesting that patients place significant importance on continuity and provider accessibility during critical moments. These expectations reflect patients’ desire for consistent support and may indicate trust in specific physician-patient relationships rather than generic service delivery [28, 29]. Experienced professionals may not have the opportunity to provide consultations outside of their working hours, so the availability of a counseling team and online counseling can be helpful.

While physician gender was rated as important by a majority, it was not among the top-ranking factors, highlighting that other dimensions—such as behavior, communication, and accessibility—may override gender preferences in the actual decision-making process. This is consistent with evidence that patient preferences in OB/GYN care are shaped by complex interplays of personal comfort, cultural norms, and perceived competence [9, 30–32]. In Iran, male medical students are not allowed to study obstetrics and gynecology, and only a small percentage of specialists in this field are male. Some elderly male specialists still practice medicine and are highly regarded by certain patients for their reputation and history.

We found that recommendations from friends, family, and peer physicians/colleagues, as well as a physician’s reputation, were moderately important factors for patients in choosing an obstetrics and gynecology physician. Previous research has suggested that a physician’s reputation and recommendations from peers can act as a substitute for their expertise, skill, and experience [33].

Importantly, this study moves beyond a consumerist model of patient behavior by framing preferences as expressions of patients’ needs for respectful, responsive, and ethically grounded care. Word-of-mouth recommendations, for instance, can be interpreted not as marketing outcomes but as reflections of trust and satisfaction transmitted through social relationships.

Our study revealed that patients belonging to different groups had varying non-clinical preferences. Those referred to multiple specialist physicians placed importance on respect and attention from the physician and staff, out-of-hours availability, information about office hours and physician’s presence, and office facilities. More than 60% of participants fell into this category, which could indicate an effort to make informed choices and find the ideal physician.

The influence of education level and pregnancy experience on preferences points to variation in expectations across demographic groups. Patients with higher education tended to value insurance coverage, para-clinical proximity, and comfort-related amenities more, possibly reflecting greater health literacy and awareness of healthcare navigation. These nuances reinforce the need for differentiated care strategies tailored to patient backgrounds [34]. Making an informed choice can lead to higher quality services, positive evaluations, and patient satisfaction [35].

The study found a significant relationship between pregnancy experience and patients’ concerns about insurance coverage and surgery/childbirth costs. This highlights the financial concerns of patients regarding treatment costs in hospitals with physician contracts [36]. In Iran, primary health insurances typically cover only one-third of the treatment costs, with supplementary medical insurance covering the remaining costs. However, some specialist physicians refuse to accept patients’ insurance due to challenges with tax conditions and insurance organization bureaucracies. Therefore, additional costs for diagnosis, para-clinical services, and surgeries become important for most patients, influencing their choice of specialist physician and hospital type [7, 33]. Hospitals’ hoteling facilities can also influence patients’ selection of a specialist physician for surgery and childbirth.

Patients also valued the availability of information about para-clinical services, their proximity to the office, and office amenities such as a spouse’s room and lactation room. The physical conditions of patients and long waiting times emphasize the need for more comfort and amenities in obstetrics and gynecology offices compared to other specialties. This has been highlighted in previous studies as a necessary factor for patient satisfaction and medical marketing. [20, 25, 37].

In this study, university affiliation (being a university faculty member), physician’s age, and proximity of the office to the home were the least important parameters in choosing a physician, which is consistent with previous studies [6, 8, 31, 38, 39]. Although it contradicts with other studies [40–42].

This study’s cross-sectional design and focus on private practices in one city limit generalizability, and further research in different specialties and settings would be valuable. Nonetheless, the findings offer a strong foundation for improving patient-provider interactions, informing clinic policies, and guiding educational efforts in communication and ethics.

## Conclusion

The OB/GYN private practices, delivering ethically grounded, patient-centered care involves more than clinical competence-it requires attentiveness to the non-clinical aspects of care that matter most to patients. Respect, communication, privacy, and accessibility emerged as key priorities for women when choosing a provider, reflecting their desire for care that is responsive, trustworthy, and emotionally supportive.

By understanding and integrating these preferences, healthcare providers and institutions can foster stronger relationships, enhance patient satisfaction, and support shared decision-making. These outcomes are not only markers of quality but are also central to ensuring respectful and dignified care for women in sensitive healthcare contexts.

The findings of this study offer practical guidance for clinicians, administrators, and policymakers seeking to align service delivery with patient values—particularly in private sector settings where patient choice plays a significant role in care-seeking behavior.

## Data Availability

The data used and analysed during the current study are not publicly available due Mashhad University of Medical Sciences policy, but are available from the corresponding author on reasonable request.

## References

[1] Kelley SW, Schwartz RW. A marketing-oriented perspective on physician selection. Surg Innov. 2005;12(4):357–63. 16424958 10.1177/155335060501200412

[2] Magnezi R, Bergman LC, Urowitz S. Would your patient prefer to be considered your friend? patient preferences in physician relationships. Health Educ Behav. 2015;42(2):210–19. 25156313 10.1177/1090198114547814

[3] Cooley DO, Madupu V. How did you find your physician?: an exploratory investigation into the types of information sources used to select physicians. Int J Pharm Healthc Mark. 2009;3(1):46–58.

[4] Kim K, et al. Factors associated with patients’ choice of physician in the Korean population: database analyses of a tertiary hospital. PLoS One. 2018;13(1):e0190472. 29293614 10.1371/journal.pone.0190472 PMC5749849

[5] Chen A. Understanding patient choice: a study of women’s choice in prenatal screening and testing. 2017.

[6] Shamrani H. A cross-sectional survey of Women’s provider gender preferences for gynecology and obstetrics Care at King Abdulaziz University hospital. J Women’s Health Care. 2016;5(347):2167–0420. 1000347.

[7] Kuruoglu E, et al. Which family physician should I choose? The analytic hierarchy process approach for ranking of criteria in the selection of a family physician. Vol. 15. BMC Medical Informatics and Decision Making; 2015. p. 63. 10.1186/s12911-015-0183-1 PMC4525740 26242399

[8] Groutz A, et al. Do women prefer a female breast surgeon? Isr J Health Policy Res. 2016;5(1):35. 27980717 10.1186/s13584-016-0094-3 PMC5131538

[9] Willis E, et al. Women and gynaecological cancer: gender and the Doctor-patient relationship. Topoi. 2017;36(3):509–19.

[10] Emmert M, Sander U, Pisch F. Eight questions about physician-rating websites: a systematic review. J Med Internet Res. 2013;15(2):e24. 23372115 10.2196/jmir.2360 PMC3636311

[11] Chang D-S, Chen W-L, Wang R. Impact of the bidirectional relationship between communication and cognitive efficacy on orthopedic patient adherence behavior. BMC Health Serv Res. 2022;22(1):199. 35164761 10.1186/s12913-022-07575-5 PMC8845262

[12] Keating NL, et al. How are patients’ specific ambulatory care experiences related to trust, satisfaction, and considering changing physicians? J Gener Intern Med. 2002;17(1):29–39. 10.1046/j.1525-1497.2002.10209.x PMC1494999 11903773

[13] Tahmasebian S, Ghazisaeedi M, Langarizadeh M, Mokhtaran M, Mahdavi-Mazdeh M, Javadian P. Applying data mining techniques to determine important parameters in chronic kidney disease and the relations of these parameters to each other. J Renal Inj Prev. 2016;6(2):83–7. 10.15171/jrip.2017.16. PMID: 28497080; PMCID: PMC5423289. 10.15171/jrip.2017.16 PMC5423289 28497080

[14] Setoodefar M, et al. Measurement Model of Women’s preferences in Obstetrician and Gynecologist selection in the Private sector: exploratory and confirmatory factor analysis. Int J Community Based Nurs Midwifery. 2020;8(2):150. 32309456 10.30476/IJCBNM.2020.82278.1049 PMC7153421

[15] Norman G. Likert scales, levels of measurement and the “laws” of statistics. Adv Health Sci Educ. 2010;15(5):625–32. 10.1007/s10459-010-9222-y 20146096

[16] Alkuwaiti A, Maruthamuthu T, Akgun S. Factors associated with the quality of outpatient service: the application of factor analysis-A case study. Int J Healthc Manag. 2020;13(sup1):88–93.

[17] Hughes F, Bernstein PS. Sexism in obstetrics and gynecology: not just a “women’s issue". Am J Obstet Gynecol. 2018.

[18] King D, et al. Identifying quality indicators used by patients to choose secondary health care providers: a mixed methods approach. JMIR Mhealth Uhealth. 2015;3(2):e65. 26048441 10.2196/mhealth.3808 PMC4526909

[19] Gholami Fesharaki M, et al. Inpatient satisfaction and effecting factors: findings from a large sample size cross sectional study. Health Res J. 2016;1(1):1–2.

[20] Perrault EK, Inderstrodt-Stephens J. Us mothers’ behaviors and preferences when choosing physicians for their families. Assisting “Chief Med Officers”. Health Care For Women Int. 2017;38(11):1234–46. 10.1080/07399332.2017.1367002 28825522

[21] Zodan T, von Orelli S. Teaching communication in an emergency gynecological setting. Cogent Med. 2018;1491092.

[22] Naimer MS, et al. Specialist wait time reporting using family physicians’ electronic medical record data: a mixed method study of feasibility and clinical utility. BMC Primary Care. 2022;23(1):1–14. 10.1186/s12875-022-01679-x PMC8988329 35392824

[23] Zykienė B, Kalibatas V. Evaluating the reasons for nonattendance to outpatient consultations: is waiting time an important factor? BMC Health Serv Res. 2022;22(1):1–9. 35534875 10.1186/s12913-022-08033-y PMC9082880

[24] Mazaheri Habibi MR, et al. Evaluation of patient satisfaction of the status of appointment scheduling systems in outpatient clinics: identifying patients’ needs. J Adv Pharm Technol Res. 2018;9(2):51–55. 30131937 10.4103/japtr.JAPTR_134_18 PMC6078003

[25] Devlin AS. Seating in doctors’ waiting rooms: has COVID-19 changed our choices? HERD: Health Environ Res Des J. 2022;19375867221104248. 10.1177/19375867221104248 35726212

[26] Rogo-Gupta LJ, et al. Physician gender is associated with press Ganey patient satisfaction scores in outpatient gynecology. Women’s Health Issues. 2018;28(3):281–85. 29429946 10.1016/j.whi.2018.01.001

[27] Shahawy S, Deshpande NA, Nour NM. Cross-cultural obstetric and gynecologic care of Muslim patients. Obstet Gynecol. 2015;126(5):969–73. 26444117 10.1097/AOG.0000000000001112

[28] Knight HE, et al. Birth “out-of-hours”: an evaluation of obstetric practice and outcome according to the presence of senior obstetricians on the labour ward. PLoS Med. 2016;13(4):e1002000. 27093698 10.1371/journal.pmed.1002000 PMC4836717

[29] Kashgary A, et al. The role of mobile devices in doctor-patient communication: a systematic review and meta-analysis. J Telemed Telecare. 2017;23(8):693–700. 27632990 10.1177/1357633X16661604

[30] Sharif Nia H, et al. Development and psychometric evaluation of a Persian version of the death depression scale-revised: a cross-cultural adaptation for patients with advanced cancer. Jpn J Clin Oncol. 2017;47(8):713–19. 28505271 10.1093/jjco/hyx065

[31] Amer-Alshiek J, et al. Israeli Druze women’s sex preferences when choosing obstetricians and gynecologists. Isr J Health Policy Res. 2015;4(1):13. 26034576 10.1186/s13584-015-0013-z PMC4450487

[32] Johnson AM, et al. Do women prefer care from female or male obstetrician-gynecologists? A study of patient gender preference. J Am Osteopath Assoc. 2005;105(8):369–79. 16166391

[33] Yahanda AT, et al. A systematic review of the factors that patients use to choose their surgeon. World J Surg. 2016;40(1):45–55. 26362880 10.1007/s00268-015-3246-7

[34] Robertson R, Burge1 P. The impact of patient choice of provider on equity: analysis of a patient survey. J Health Serv Res Policy. 2011;16(1_suppl):22–28. 10.1258/jhsrp.2010.010084 21460346

[35] Wahlstedt E. The impact of internet based information sources on patient satisfaction of care in region skåne-A cross sectional study using survey data. 2014.

[36] Emmert M, Halling F, Meier F. Evaluations of dentists on a German Physician rating Website: an analysis of the ratings. J Med Internet Res. 2015;17(1):e15. 25582914 10.2196/jmir.3830 PMC4319074

[37] Liu N, et al. When waiting to see a Doctor is less irritating: understanding patient preferences and choice behavior in appointment scheduling. Management Science; 2017.

[38] Amir H, et al. Bedouin Women’s gender preferences when choosing obstetricians and gynecologists. J Immigr Minor Health. 2016;1–8. 10.1007/s10903-016-0522-z 27796701

[39] Augustin J, et al. Analysis of patients’ willingness to be mobile, taking into account individual characteristics and two exemplary indications. J Dtsch Dermatol Ges. 2017;15(4):430–38. 28314064 10.1111/ddg.13218

[40] Al-Briek A, et al. Factors that influence patients in choosing their treating physicians in the Private sector in Saudi Arabia. Am J Public Health. 2018;6(4):173–81.

[41] Mosadeghrad T. Effective factors in choosing a doctor in Tehran. Sci J Med Organ Of The Islamic Repub Of Iran. 2017;32(4):337–47.

[42] Kiani B, et al. Revealed access to haemodialysis facilities in northeastern Iran: factors that matter in rural and urban areas. Geospat Health. 2017;12(2). 10.4081/gh.2017.584 29239556

